# Outdoor Recreation for Older Adults in Scotland: Qualitatively Exploring the Multiplicity of Constraints to Participation

**DOI:** 10.3390/ijerph18147705

**Published:** 2021-07-20

**Authors:** Margaret Currie, Kathryn Colley, Katherine N. Irvine

**Affiliations:** Social, Economic and Geographical Sciences Department, The James Hutton Institute, Craigiebuckler, Aberdeen AB15 8QH, UK; kathryn.colley@hutton.ac.uk (K.C.); katherine.irvine@hutton.ac.uk (K.N.I.)

**Keywords:** active ageing, hierarchical leisure constraints model, outdoor recreation, qualitative vignettes

## Abstract

Active ageing can lead to better health outcomes in older people. Examining constraints to outdoor recreation for older people, including outdoor physical activity, may therefore assist with developing strategies for active ageing. Findings are presented from a study seeking to understand the constraints to older peoples’ access to outdoor recreation in Scotland, and this paper aimed to examine the multitude of constraints that discourage or prevent older people from accessing the outdoors and the ways in which these constraints are hierarchical (or not). This paper adopted a qualitative methodology using the hierarchical leisure constraints model (HLCM) as a lens to analyse the data, presenting the data in three vignettes. The paper identified multiple co-occurring constraints and considered these in relation to expectations based on the HLCM. Recognising that constraints to outdoor recreation for older people are multiple, co-occurring and mutually reinforcing may enable more effective solutions to be developed to overcome them.

## 1. Introduction

Natural settings are places that can promote health and wellbeing [1,2] and, as such, serve as spaces to promote physical activity, mental health and social interaction [3]. Living near such spaces is associated with lower mortality rates in some groups of people [4,5]. Zhang et al. [6] state that environmental benefits can counteract urban threats and improve peoples’ health outcomes, whilst daily greenspace exposure has also been found to directly affect people’s physical activity [7]. Caloguiri and Elliot [8], however, warn that the link between natural environments and health is complex and not yet fully understood. Indeed, Irvine et al. [9] argue that inventions that seek to promote health and wellbeing through contact with the natural environment should be conceptualised as a complex intervention.

We use the term “outdoors” in this paper to refer to natural or semi-natural open spaces and landscapes, from urban public parks to agricultural and wilderness environments [10]. We argue that outdoor space is as important with regards to how people use it as the natural features within it and, as such, assume that all outdoor spaces, as defined above, may have the potential to have a beneficial (but not necessarily equally beneficial) impact on health and well-being. The term “greenspace” is not used due to the fact of its more urban inference, as this study also included coastal and rural contexts.

Research reports that policy changes in the UK state that everyone should have access to good quality greenspace near to where they live [11,12]. People undertaking green exercise (i.e., physical activity in natural spaces) have the potential to save society money, and knowing why people choose to engage in green exercise (or not) could help inform how it can be more effectively promoted [8]. Dallimer et al. [12] argue that policy should focus on promoting access to outdoor spaces by those currently not using it as well as encouraging infrequent users to use it more often. There is thus need for evidence on how different groups of people use, as well as engage with, the outdoors, and factors, such as gender, ethnicity, socio-economic status and age, can all affect engagement and recreation in the outdoors [2]. Reasons for differences in outdoor engagement include the frequency by which social interactions take place [13] and the affordability of being able to make such interactions [14] but may also be more complex such as how different people feel when they are in the outdoors. Further, motivations, attitudes and values attached to outdoor engagement vary among social groups [2], which are important to consider when seeking to understand how such spaces promote health [15].

Older age groups are less likely to access outdoor environments for recreational purposes than younger or middle-aged groups of people [16,17]. Outdoor spaces may be important for healthy ageing, which refers to optimising opportunities for good health so that older people can take an active part in society and enjoy an independent quality of life [18] including retaining physical and psychological function [19] and independence and quality of life [20]. While physical activity in older adults may prevent the development and exacerbation of some conditions, such as type 2 diabetes, heart disease and arthritis, physical activity has been found to decline with age, particularly sharply for those over the age of 80 [21]. It has been argued that outdoor recreation may be the best source of physical activity for older people, as it is easy to incorporate into everyday life [7,22], but this may be easier for older people living in the greenest neighbourhoods [19] or if the built environment has certain age-friendly features [20]. Activity in the outdoors has been shown to enhance both the health and quality of life of older people [23,24] and to promote social interactions [25,26]. However, it has been found that as people become older (particularly the ‘oldest old’), their social networks decrease [27]. Decreased social networks can increase experiences of loneliness and depression [28]. An inability to engage in activities outside the home has been found to have a negative impact upon an older person’s well-being [26]. 

When considering how the benefits of being in outdoor settings can be achieved in later life, it is also helpful to think about the different barriers or constraints that exist that hinder that participation. Barriers can be considered as those factors that result in non-participation in outdoor recreation, whilst constraints may inhibit participation in outdoor recreation. Much work on facilitating access to leisure opportunities has now opted for the use of the word constraints over barriers, as constraints should be able to be overcome or negotiated, i.e., there is a solution that should result in participation in an activity [29]; constraints were thus adopted as the preferred term in this paper. Outdoor recreation is one of the most common ways in which older people engage with the natural environment [20] and can be viewed as a type of leisure activity. In this paper, we employed the hierarchical leisure constraints model (HLCM) as a lens to consider the constraints that older people face in undertaking outdoor recreation. The model proposes that constraints to leisure are arrayed in a hierarchical fashion at the individual (intrapersonal), interpersonal and contextual or structural levels and that interactions exist between factors on the three levels. The model was intended to be a universal framework that could be used to explain (non-)participation of all leisure behaviour participation and states that leisure constraints exist in these three levels which must be overcome sequentially. It theorises that where multiple constraints to leisure participation exist, attempts to negotiate constraints at the contextual or interpersonal level will be insufficient to promote participation without first addressing constraints applying at the intrapersonal level [30]. Through the application of a qualitative approach, we responded to the call by Godbey et al. [30] for research to “go beyond simply describing or classifying leisure constraints to the more challenging process of understanding how they are formed” (p. 118).

In this paper, we argue that it is important to examine constraints to outdoor recreation for older people, as it may assist with developing strategies for active ageing. This paper presents findings from a study seeking to understand the constraints to older peoples’ participation in outdoor recreation in Scotland, UK, through the lens of the HLCM. We aimed to explore the ways in which the constraints to outdoor recreation for older people are multiple and co-occurring. A qualitative approach was used to understand the nuance of multiple constraints, and we used vignettes that sought to present a holistic view of the experience of our selected participants. We used these findings to critique the usefulness of the HLCM for developing an understanding of how multiple constraints to outdoor recreation are experienced and negotiated in practice by older people.

## 2. Methods

### 2.1. Research Design

A qualitative methodological approach was employed to enable people, through their own stories, to explain the ways in which constraints to outdoor recreation occurred [31]. Qualitative approaches have been advocated by others for providing clearer insights into the experiences of older people and their engagement with natural settings [32]. A multiple case study design was adopted [33], to include participants spanning different social backgrounds and different places varying in urbanity, with access to a range of different types of outdoor settings. Three study areas in the north and east of Scotland, UK, were selected: one urban area, one coastal town and one rural area.

### 2.2. Participants and Recruitment Process

Twenty-seven individuals (19 female) participated in the study (Table 1). Participants ranged in age from 66–91 years old. A three-fold approach to recruitment was adopted. Firstly, visits were made to local community organisations running activities for older people. These organisations included local charities providing lunch clubs and social activities, a church-based craft group and local health walks groups. Secondly, notices were placed in public places and in local newspapers. Thirdly, snowball sampling was employed whereby potential participants were introduced or put in contact with the researchers through contacts in community organisations and/or participants already engaged in the research.

Ethical approval was granted by the James Hutton Institute’s Research Ethics Committee (number 45). Inclusion criteria were added to the recruitment which took account of ethical considerations alongside other research objectives. Recruited participants were: (1) aged 65 years or over; (2) retired or economically inactive; (3) living independently (e.g., not a resident of a residential or care home); (4) a year-round resident of the study area; (5) able to give informed consent for participation. To verify the latter criterion, a brief conversation was held with each potential participant prior to the interview to assess that they understood what the interview was about and their rights as a participant. Efforts were made to maximise variety in the sample by obtaining as close to a balance in gender as possible and ensuring that participants varied in terms of age and physical abilities in each of the selected case study areas.

### 2.3. Data Collection and Analysis

Data were primarily collected through semi-structured interviews; in the rural case study, one focus group format was used at the request of six participants who felt more comfortable having a group discussion. Both data collection formats focused on the following topic areas: engagement with the outdoors for recreational purposes both now and at other points of their lives; perceived constraints to outdoor recreation; ways to facilitate outdoor recreation and physical activity. The majority of interviews took place face to face, either in the participant’s home, in a public place (e.g., local café) or at community facilities. Two interviews in the rural case study were conducted by telephone at the participants’ request. All data were audio recoded, with written (or for telephone interviews, recorded orally) consent, and transcribed for analysis.

Transcripts were analysed through a process of in-depth thematic analysis [34], carried out by the two interviewers (MC, KC). An initial coding framework was developed in discussion between the interviewers, with further codes added as these emerged in the analysis; thus, we incorporated both a researcher-anticipated (etic) and participant-derived (emic) approach to data analysis [35]. One interview was coded by both interviewers to ensure consistency in the coding approach, with transcripts coded separately thereafter. Insofar as possible, each interviewer coded interviews conducted by the other researcher to ensure that both had an in-depth grasp of the full breadth of data.

Constraints to use of the outdoors were an etic theme for which the data were analysed, then the emic theme of “multiple and co-occurring constraints” arose. The data were then systematically analysed a second time, specifically introducing these multiple co-occurring interactions as a new etic theme and analysing these through the lens of the HLCM [30]. The aim of this process was to explore the connections between constraints and the extent to which they were negotiated in a hierarchical fashion. Our analysis sought to identify the reasons for and relationships between multiple constraints in the data. Participants did not necessarily explicitly reflect on this multiplicity, and it was revealed by looking holistically at each person’s experiences, rather than through discrete quotes, hence, the decision to present the data in vignettes in the results. The use of vignettes allowed us to bring the themes together into narratives with the inclusion of some direct quotes. All the vignettes were pseudonymised. A similar approach of using vignettes was employed by [33] when considering the factors that facilitate physical activity in older adults. Such a phenomenological presentation of the data allowed us to present both what was being experienced and how it was experienced [36] and was thus felt to be the best way to highlight the way in which the experienced constraints were multiple.

## 3. Results

The results are presented in two sections. The first (Section 3.1) explores the different constraints that participants attributed to preventing outdoor recreational opportunities from occurring. The second (Section 3.2) reflects on the multiplicity of these constraints through the lens of the HLCM using three vignettes to illustrate. 

### 3.1. Individual Constraints

Nine key constraints were identified across the interviews. Table 2 organises these individual constraints by whether they align with individual (intrapersonal), interpersonal or structural levels of the HLCM. Exemplar quotes are used to provide context. 

The intrapersonal constraints (i.e., those particular to the individual) were thus identified as individual health and mobility problems, fragility and vulnerability and lack of motivation and constraining attitudes. Identified interpersonal constraints (i.e., those constraints caused by others affecting the individual) were safety concerns, social barriers as well as the health and mobility of others. Structural barriers were time commitments, access to outdoor spaces and weather and daylight; whilst the latter were external to the individual, they were nevertheless constraining. Gender was also identified as a cross-cutting theme, it was felt that it affected all situations people were in and decisions they made, and for this reason was felt to cut across intrapersonal, interpersonal and structural constraints. As examples of gender’s influence across the different levels, at the intrapersonal level gender was found to affect motivation; at the interpersonal level gender affected feelings of safety; at the structural level, gender affected the ability to access outdoor spaces.

### 3.2. Multiple Co-Occurring Constraints

Participants rarely spoke about constraints in isolation but instead spoke of numerous constraints that reduced or prevented them from accessing outdoor recreation opportunities. The way in which constraints co-occurred and the extent to which they were hierarchical in type are explored by the three vignettes in this section. 

#### 3.2.1. Vignette 1 (Jenny)

Jenny (73) lives with her husband in a large urban area in Scotland where they have spent most of their lives. She used to thoroughly enjoy outdoor recreation but broke her ankle a few years ago which required plates and pins to fix it, and it still gets stiff and sore. Because of this injury, Jenny does not want to walk further than a mile at a time. Jenny also states that she is busy: “I always seem to have something else on”. Jenny used to be part of a friendship group, but that stopped when the group ended. Jenny says that sometimes she goes out for a walk with a neighbour but “not so much this year because her husband’s been ill”.

Jenny describes multiple constraints that include social (not having someone to go out with); finding the time in a busy (perceived or otherwise) schedule; health of others; safety; time of day (and possibly of year); motivation (as illustrated in Box 1). She talked about the appropriateness of footpaths, made worse when it is raining, for example, “Well, I wouldn’t walk if it was pouring rain…and if it’s slippy under foot, I’m very wary of...you know, since I broke my ankle [little laugh]”. Box 1 provides an extract from the interview to highlight the different considerations she makes if contemplating undertaking outdoor recreation.

Jenny’s constraints are multiple. These constraints include health and a lack of motivation (intrapersonal), not having someone to go with and safety (inter-personal), other time commitments and distance to access an outdoor recreation opportunity (structural). For Jenny, the numerous constraints she faces seem to collectively act to deter her from going outside, i.e., the constraints appeared to mutually reinforce each other. Mostly these constraints do not appear to act in a hierarchical fashion—as overcoming one does not overcome another. One exception, however, is that having someone to go with does seem to overcome the safety constraint, which may also allow her to justify the time commitment due to the socialising opportunity it presents.

Box 1 **Extract from Jenny’s interview**

Interviewer: Would you ever choose to…go for a walk around a park or...Jenny: Yes, well...sometimes...you know, I live near [large local] Park, and my neighbour and I have had a walk when the summer nights... Not so much this year because her husband’s been ill... But ehm, we used to do that last year in the evening—have a...walk around the park and home again...but she was very slow because she’s...she’s had two knee replacements, so [little laugh]Interviewer: RightJenny: I mean it was more....it wasn’t really...I would say exercise as such, you know, when you’re walking fast to try and.eh, keep your heart beat up and that. But…I used to do that. But as I say, this year her husband’s been ill and she hasn’t been able to do it...Interviewer: So, would that be something that you would only kind of do if you had company, like say your neighbour...Jenny: YesInterviewer: or would you go yourself?Jenny: I don’t think I would go myself...eh, through the park...Interviewer: Why would that be?Jenny: I don’t know...You hear folk, you know...I wouldn’t do it at night certainly...You know, if it was light during the day I probably would, but it’s getting the time—[laughing] I’ve got other things on during the day!Interviewer: Would you feel safe if you went on your own during the day do you think?Jenny: Probably yes…Interviewer: Yeah. So, it’s not that you’re...necessarily put off of going...Jenny: NoInterviewer: you’ve just...Jenny: It’s just getting...fitting in with all the other things I try to do! [laughing]

#### 3.2.2. Vignette 2 (George)

George is 74 and lives with Alison, his wife, in a small town in a National Park in Scotland; it is a place many people would choose to go on holiday. George has lived in the area for less than twelve years. He and Alison moved there when they retired after repeated holiday visits throughout their lives. George and Alison thought that living in the area would be like being on holiday all the time, but “being on holiday somewhere is different from living there” because, he says, everyday life, such as housework and keeping the garden, right “gets in the way”. He says he now does not prioritise going outside as much as he used to mainly because of time constraints. He currently volunteers for the church and helps out with his daughter’s animals and his neighbour’s dog.

When George first moved to the area, he frequently went cycling with a group of other people, but they have all since moved away. George and Alison also went skiing with other friends, but they also moved away and Alison also has health issues stopping this now too. 

George and Alison are members of more than one local walking group. He enjoys the social aspect of the group, but he has not been going as much recently because he had cancer three years ago and, although it has been treated, he finds he tires easily and the walk is further than he can manage, or he feels he can only manage if the walk starts and finishes in the same place. Health issues have changed his priorities. He could probably manage, he says, but does not do it anymore. On top of this, he finds himself as one of very few men in the group. He thinks that women and men tend to feel differently as they age—“women congregate, men tend to isolate themselves”—which makes forming new social networks with “like-minded people” more difficult. 

Many of the constraints that George faces act collectively to decrease participation in outdoor recreation. The predominant constraints identified are time (structural), not having someone to go with (intra-personal), health (inter- and intra-personal), but there also exists an over-arching gender constraint. When entering later life, George was found to be initially highly motivated to access the outdoors for recreational purpose; however, George and Alison have found living in the locality different to visiting it on holiday, as everyday life brings with it additional time commitments. The time constraint appears to act more independently to others which reinforce each other. Both George and Alison now have health problems that limit their abilities to participate in activities they once did, and George’s social connections previously facilitated his ability to ski and cycle, but these no longer exist. Alison did not cycle, but they previously both skied together which they can no longer physically do. As George has aged, his health has led him to be less physically able which has shifted his habits and priorities away from outdoor recreation opportunities. George and Alison also feel it is difficult to expand their social circle and undertake new opportunities. The local walking group did expand their social circle but George’s current health difficulties mean that he cannot walk as far or as easily as he once could with the group and he feels he gets less out of it. On top of this, he feels that there are more women in the group and states that his gender deters his participation, as he feels like he has less in common with the rest of the group being the only man. 

#### 3.2.3. Vignette 3 (Betty)

Betty lives with her husband, John. She is in her mid-60s and was originally from the case study area. Betty is quite socially active and frequently participates in the local walking group (more than once a week). Due to ill-health, John is housebound, and Betty does not get out as much as she would like to. She used to go with John, and she enjoys the company. 

Betty discussed a time approximately 18 months prior to the interview when she was followed by someone when she went for a walk near a local graveyard. She ran away from them and had to climb over a wall. This experience scared Betty and, since then she has been experiencing panic attacks as well as other health problems including shingles. Before this experience, Betty stated that she really enjoyed going outside for walks, but now she does not want to go out alone. This is an improvement from immediately after the experience when Betty did not want to go out at all. 

There are other constraints that prevent Betty from getting out. When Betty is interviewed it is mid-November and the rain pounds down on the roof under which we are sitting. It is 4 o’clock and getting dark. Betty talks about it being more difficult getting out at this time of year anyway, because it gets dark early. This means that there is less time in the day to fit in walks and because the dark fuels Betty’s fears of being followed again. Betty went to see a doctor about the panic attacks, who diagnosed her with high blood pressure and prescribed Betty medication for the panic attacks; this has helped her overcome her fear of getting out. 

Betty says that other people have encouraged her to get out, but she does not want to go out alone. She was encouraged by friends to join a local walking group to give her some company when participating in outdoor recreation. She has found that since her walking has increased, her blood pressure has gone down. Joining the walking group has facilitated her getting out, but the rain and cold still stop her wanting to go out. She also finds that because the walking group is regular, it is possible to fit it into her already busy routine of helping out with John at home and her grandchildren after school. Betty does think that there may be a time that she will get into the outdoors more and maybe by herself, but not just now. She does not ever think that she will go back to the place where she was followed by herself, although she has gone back with company. This prevents her from accessing her most local greenspace alone. 

The constraints that Betty faces are multiple and together interact to decrease her participation in outdoor recreation. The predominant constraints identified are health, not having someone to go with and safety (inter-personal) and time and weather and daylight (structural). These constraints, however, do not appear to be hierarchical, but their plurality and co-occurrence mutually reinforce each other and affect her motivation (inter-personal) and ability to seek outdoor recreation opportunities. Betty is unmotivated to go out when it is dark or when the weather is inclement. Not going out in the dark and bad weather could be argued to be motivational but, since she was attacked, Betty is more worried and, therefore, her lack of participation includes safety constraints. Betty’s attack has deterred her from seeking out outdoor recreation opportunities alone, whereas she did before the attack. Until recently, Betty’s husband was the person who she was most likely to go out with, but he is now housebound. Her husband being housebound adds to her time constraints, in addition to caring responsibilities for her grandchildren before and after school. She is more motivated to undertake outdoor recreation when it fits in with a routine and she can personally negotiate it into her schedule. The walking group facilitates her participation in outdoor recreation as it fits in with her routine and she has friends she can go with. Her improved blood pressure (which she attributes to participation in the walking group) and not letting her friends down/her expected attendance at the group further motivates her.

## 4. Discussion

This paper responded to calls to understand the nuance of multiple and co-occurring constraints to outdoor recreation in older people. Dalton et al. [30] found that limiting long-term conditions can exacerbate difficulties in maintaining social connections [30]. Other studies can be found on constraints to physical activity by older people that may also be relevant to outdoor recreation [20]. Examples include Baert et al. [21], who found that participation was a dynamic and complex process and influenced by various factors, e.g., a lack of interest, fear of falling, (lack of) social support, and environmental factors, health conditions, being overweight, poor balance, muscle weakness and shortness of breath, lack of interest and motivation. Franke et al. [22] identified additional barriers, including insufficient time; lack of facilities; transport and money; poor health; fatigue; lack of interest; lack of company; enjoyment/knowledge; inclement weather; adverse environments; injury; joint pain; a perception of being too old concluding that “there is a great need for more studies that address the influence of the social, built and natural environments (and the intersections between them) on older adult behaviours including physical activity” (no page number). Baert et al. [21] also agreed constraints need further investigation, specifically intrapersonal and community factors, as ways to overcome them can be facilitated by policy. This paper furthers understanding about constraints by finding that constraints experienced by older people should be acted on holistically and not in isolation as numerous constraints act together and need to be responded to as such.

In this paper, constraints to the use of outdoors for recreational purposes amongst older people living in three different types of settings—cities, rural towns and coastal towns—are qualitatively examined adding to calls by others to explore links between ageing, recreation and nature and to provide more nuance to understandings [21]. Freeman et al. [32] argue that “only by understanding the relationship between nature, ageing and the physical environment can more age-friendly places be developed…for many older people the barriers to accessing such spaces can be insuperable…barriers to accessing the outdoors are not confined to rest home residents but similarly impact on older adults who are ageing at home…but also experiencing declining health and mobility and adverse or stressful life events such as loss of a spouse” (p. 2). In their study, they explored the impacts of ageing on health conditions, the loss of mobility on nature connections, the ways older adults respond to these changes and identify the types of nature engagements and greenspaces that are prioritised and responded to by older adults; this study builds on these understandings [32] by examining the complexity and interactions of multiple constraints in hindering outdoor recreation. Stephens et al. [37] identified that older peoples’ capabilities are limited by their social and material circumstances and state that the current healthy ageing discourse places responsibility on individuals for achieving good physical health and ignores their broader circumstances, our findings support the suggestion that broader circumstances are important and can hinder older peoples’ participation in outdoor recreation. Phoenix and Bell’s [38] study examined the physical, social and cultural environments that support and hinder participation in physical activity in older people to provide a nuanced insight into the complexities that are inherent to being physically active in the latter half of life; this paper, with its qualitative design, adds to these papers by having a more nuanced insight into complexities of outdoor recreation participation. 

Relationships between older people and their engagement with natural environments are thus complex, and our findings support that a lack of understanding about these engagements limits the effectiveness of interventions which support active ageing [19]. Part of the complexity is that there are multiple pathways through which natural outdoors settings influence health in adults [2] and use of outdoor space should be seen as a process, not an event, that involves many interwoven factors [39]; we would add that the constraints that any individual experiences should be considered and addressed together rather than separately. These factors link to pathways which include personal, social and environmental factors that some [19,40] argue have not been well evaluated. Our findings identified that constraints are multiple and co-occurring and should be considered as such by interventions if the interventions are to be effective. Specifically, the paper has sought to explore the ways in which constraints are multiple and has examined the relevance of the HCLM when applied in this (qualitative) context. All three levels of the HLCM were found in the constraints to outdoor recreation experienced by the older participants in our sample.

### 4.1. Intrapersonal Level

The constraints identified at the intrapersonal level were health and mobility, fragility and vulnerability, lack of motivation and negative attitudes. Astell-Burt et al. [41] found that worries about the consequences of falling among older adults may make venturing out in greener neighbourhoods less attractive therefore increasing the impact of social isolation on mental health. Freeman et al. [32] found that older peoples’ health affected their ability to access nature. They noted not being able to undertake strenuous activity, that their changed life circumstances included slowing down, loss of independence, being unable to do activities, change in holidays and that a partner’s poor health can constrain the ability to go out; thus, Freeman begins to address multiple co-occurring constraints faced by older people. We further explore this concept explicitly and highlight that multiple co-occurring constraints are acting holistically to deter outdoor recreation participation in older adults in our sample.

### 4.2. Interpersonal Level

The constraints identified at the interpersonal level were social, safety and other people’s health. Kemperman and Timmermans [42] argue that older people may miss out as they have fewer social networks and spend more time indoors. Outdoor spaces have been found to strengthen a sense of community via place attachment and place identity [40], for example, Hawkins et al. [43] found this to be the case for older people working at allotments. Hartig et al. [2] identified social cohesion and increased social contacts as a key pathway through which the natural environment supports health promotion and may even be a motivation for older people to use outdoor spaces [44]. We suggest that many other constraints act to further deter social interaction which may be necessary to facilitate outdoor recreation in older people, so although social contacts can act as a pathway to outdoor recreation, that pathway can be blocked by other constraints (e.g., caring for others, lack of time, and poor weather). Jennings and Bamkole [45] considered how outdoors spaces support social interactions and social cohesion; however, their conceptualisation did not consider whether a lack of social contacts may deter people from accessing outdoors spaces, which is what we found and, as such, older people facing constraints to outdoors spaces due to the lack of companions may also be less able to generate new social connections than those who can get outdoors. Astell-Burt et al. [41] state “social interactions which are entwined with physical activity (also) decline as we age, yet this [social interactions] is another mechanism linking greenspace with better mental health” (p. 602), which highlights the importance of considering how constraints collectively act to deter an older person to participating in outdoor recreation—a lack of participation can decrease other possible social interaction opportunities. Paddon [18] examined how walking groups may negatively influence well-being later in life. She found that social contact is not enjoyed by everyone, and it can be difficult for some people to engage with already established groups; walking groups were also found to remind some people of their declining mobility, and the dynamic does not always suit people with different needs and different abilities. Paddon’s [18] findings resonate with the experiences of George (Vignette 2). There is a need for further research that focuses on the way to facilitate social access in a number of different ways, specifically for gender and including outdoor walking groups [9,46].

Much previous research on the constraints to outdoors spaces focuses on (perceived) safety. Gatersleben and Andrews [47] found that outdoors spaces can cause fear predominantly by worry of getting lost or being attacked. Wendell et al. [48] found females in particular were constrained when places were perceived to be unsafe, which was true for the participants portrayed in the vignettes in this study. Maruthaveeran and Konijnendijk van den Bosch [49] found a fear of crime can result in anxiety and erode well-being. They argue that it is important to examine the attributes that evoke fear and how these attributes interact; this is especially important given that the origins of the fear may not be environmental but rather may be attributed to a lack of social contact in the neighbourhood. Again, this aligns with our findings which suggest that safety constraints can be overcome if people can access outdoor recreation opportunities with others.

### 4.3. Structural Level

The structural constraints identified were time commitments, access to suitable outdoor spaces, weather and daylight. Previous research has identified that older people are more mobility-impaired and have limited activity spaces [22]. Outdoor spaces within the direct living environment are particularly relevant for older peoples’ outdoor activities [50]. Others have found that good access to greenspaces may encourage higher levels of physical activity for recreation among older people [19,51]; we argue that this is only the case when other constraints have been overcome that are co-occurring to constrain participation in such activity otherwise.

Caloguiri and Elliot [8] identified a lack of time as a common barrier to physical activity, whilst Dallimer et al. [12] found that rather than socio-demographic factors, the frequency by which participants visit urban greenspace is determined by how long it takes for an individual to reach a site. This study added more nuance about time by highlighting that the older people included in this study were often busy with responsibilities, such as caring, and tended to be restricted to weekly routines.

### 4.4. Multiple and Inter-Connected Constraints to Outdoor Recreation

Previous research has identified the existence of multiple constraints that prevent older people from accessing the outdoors. Finlay et al. [24] found that for older people accessibility, costs and convenience were all constraints to accessing natural environments and that convenience and frequency of activity and passive engagement with nature impacts the physical, mental and social well-being of older adults. Whilst not specifically identifying social contacts as a constraint to accessing natural environments, they did find that local greenspaces acted as spaces to enhance social interaction, community building and empowerment. Baert et al. [21] identified many similar constraints to physical activity that we did to outdoor recreation, particularly lack of a social companion, caring for others and busyness. However, generally, they viewed social support as a motivator rather than a constraint to physical activity. Franke et al. [22] considered factors that facilitate physical activity in older adults and found the following three things: resourcefulness; social connections; built and natural environments. However, of the papers that identified a number of constraints, there has been a lack of exploration of the ways in which the multiplicity of these constraints may further decrease active participation in an activity, which is the novelty this paper provides.

The vignettes presented in this paper serve to illustrate the multiplicity of constraints experienced by participants. In vignette 1, Jenny, was selected as her story is typical of many of our participants. Vignette 2, George, was selected as it particularly highlights changing priorities with age and an interesting gender dimension and vignette 3, Betty, focuses on the impact of a major safety constraint. All the vignettes highlighted that for participants to access outdoor recreation opportunities, they need to overcome numerous constraints. It could be concluded that these findings support the preposition of the HCLM that constraints exist at different levels. However, our findings did not support that these constraints were hierarchical or interconnected in the way that the model suggests. The most important constraint for Betty is safety concerns (intrapersonal) in that if her safety concerns were overcome, she would be more likely to access outdoor recreation opportunities, but the same is not true for George and Jenny. For them, it appears that the multiplicity of constraints they face collectively contribute to a decision to participate in outdoor recreation less frequently than they might otherwise. Further, we found that gender is a cross cutting theme across all three levels of the HCLM and, as such, these findings support another study considering constraints, which employs the HCLM to explore gender, and it was found that rather than constraints being represented at three distinct levels constraints were found to be mutually reinforcing which created more complex interactions between them [52].

The HLCM has predominantly been applied to quantitative data; in this paper, we used it in a more nuanced way with qualitative data. Other studies that have applied the model using qualitative methodologies have also found interesting nuances, for example, that the model hierarchy does not always exist due to the fact of inconsistencies between the levels [53], that it may apply differently in different cultural contexts [54] and that the model cannot be applied to group activities which require simultaneous presence and, thus, reliant individuals participation is dependent on others’ abilities to participate [55]. Like these other examples, the qualitative nature of the data from this study allowed us to explore the structuring of constraints as experienced by individuals in a different way to that which would be possible through quantitative approaches.

In this study, we identified and described the nature of multiple constraints that were experienced simultaneously by older people. The results highlight that it is common for there to be more than one constraint preventing participants from using natural environments and, thus, solutions or interventions that aim to overcome one constraint may fail to produce effective change if the solution does not address other co-occurring constraints. Future research is required that considers how these findings relate to other groups of people, in other places and, thus, whether the results are replicable, and also whether novel ways in which these constraints can be overcome. Specifically, the key finding is that for older people in this study, constraints to accessing outdoor recreation opportunities do not act in isolation of each other but are multiple co-occurring; indeed, their multiplicity acts to reinforce the other constraints they face.

Efforts to get people outdoors in the UK have focused on the way such places can facilitate physical activity and mental health benefits [56]. The social value of the outdoors may be of particular importance to older people as they are a group of people known to be lonelier than other groups [26]. Our findings showed that older people wanted to participate in outdoor recreation, because the outdoors was regarded to be a social space. O’Brien and Morris [25] have highlighted the importance of social connectedness for older people in woodlands. This suggests that the outdoors should not only be seen as spaces where physical activity can occur, but also as spaces for enhancing social interaction [25,57]. Interventions should thus seek to improve how they could better appeal to different people with different motivations, e.g., both social and physical activity motivations.

### 4.5. Limitations

Our sample was a qualitative study with 27 participants which contained more female than male participants. Qualitative studies do not aim to be representative of the sample they are selected from [35] and, thus, this study should not be viewed as being representative. Previous research has noted that men are less likely than women to participate in community organisations and make less use of local health services as well as to have lower life expectancy [58]. We also note that participants had different understandings or interpretations as to what “outdoor recreation” meant. The interviewers informed participants of the definition employed in the study. Similarly, the distinction between recreation/leisure activities and other activities (such as walking to get to a destination) was not always clear to participants. For example, many participants talked about walking for active travel (e.g., walking to local shops). However, outdoor recreation was defined by participants; all forms of it involved potential benefits to health and well-being whether that be physical activity and social interaction by walking to get some local shopping or cycling in a mountain wilderness. 

## 5. Conclusions

This paper built on previous research linking the benefits of people being in the outdoors and natural environments to their health and well-being but sought to explore how older people’s participation in outdoor recreation was hindered by multiple co-occurring constraints through employing a novel application of the hierarchical leisure constraints model. The relevance of our findings to public health, specifically, are for when practitioners are thinking about interventions to promote the use of the outdoors for active ageing. They need to consider that older people face multiple and co-occurring constraints to their use of the outdoors; if these constraints are not considered as such, the interventions will be less effective in promoting positive public health outcomes for active ageing.

## Figures and Tables

**Table 1:** Case study areas and number of participants.

Case Study Area	Case Study Type	Number of Participants
1	City	9
2	Rural town	11
3	Coastal town	7

**Table 2:** Constraints and HLCM levels identified with illustrative examples.

Constraint	HLCM Level	Examples Given in the Interviews
Health and Mobility	Intrapersonal	Chronic conditions, e.g., joints being replaced (hips and knees); asthma; bronchiectasis; chest problems; osteoporosis; cancer “I’ve got a bad heart, and I’ve got a bad leg as well, but I’ve got a bad heart—I’ve got a defibrillator...” (Urban, male, 81) Acute conditions, e.g., broken bones and ligament injuries, chest infections, shingles, panic attacks and sciatica Pain F “I do get discomfort at times, but sometimes it’s...it could be in my bed, just the way I turn or... eh... I don’t know what, you know, causes it, but it’s not just my ankle, it’s maybe up the back of my leg... I think the tendons have a lot to do with it—you know, things like that. But on the whole, I’m fine, and I’m able to get out and about, so that’s the main thing” (Urban, female, 73) Tiredness Health and mobility problems of others
Fragility and Vulnerability	Intrapersonal	Fear of falling and apprehensive about being outdoors alone if this occurs “I’m very cautious because I did fall in the woods years ago and...and I broke my arm so...I do...look where I’m going!” (Rural, female, 68) Loss of confidence
Lack of motivation and constraining attitudes	Intrapersonal	Do not enjoy being outside Laziness and lack of motivations: “I’m lazy! Yeah he’s [the dog] my incentive but I’m lazy and I know I’m lazy … [later in the interview]…I need a reason and the reason I go for walks is because my doggie needs out for a walk! If he did’nae [didn’t] need out for a walk I would’nae [wouldn’t] be going out for a walk! But I’m honest about it, you get these people that say oh no I would do that anyway and I’m thinking I would’nae do nothing if I got away [with it]” (Urban, female, 76)
Safety concerns	Interpersonal	Being attacked by dogs and people
Social constraints	Interpersonal	Not having someone to go with Friends and family that they would go with being too busy, ill or having to care for others Not wanting to join a walking group without a friend
Health and Mobility	Intrapersonal	Health and mobility problems of others
Time commitments	Structural	Being too busy, e.g., volunteering, housework and gardening, having caring responsibilities “Today I’ve been for coffee, collected my grandson from the school, had lunch with him, took him back, come here, and then I’ve got country dancing after tea!” (Coastal, female, 77)
Access to outdoor spaces	Structural	Need a car to get there Cost to get there Lack of suitable spaces Poor paths, e.g., uneven ground, tree roots and mud Lack of facilities, e.g., toilets, benches and cafes Distance to access recreation opportunities
Weather and Daylight	Structural	Poor weather: “I can get out more, I just...don’t, especially not in this weather!” (Urban, Female, 66) Lack of daylight

## Data Availability

The (pseudonymised) data presented in this study are available on request from the corresponding author. The data are not publicly available to preserve the confidentiality of participants.
